# Supplementary material for: Hypersensitivity of Intrinsically Photosensitive Retinal Ganglion Cells in Migraine Induces Cortical Spreading Depression
Source: Int J Mol Sci. 2024 Jul 22;25(14):7980. doi: 10.3390/ijms25147980 (PMC11276861; doi:10.3390/ijms25147980)
Supplement: Supplementary file 1 [file ijms-25-07980-s001.zip › ijms-3113458-supplementary.pdf]

Supplementary Material

Table S1. Characteristics of the patients with migraine.

	Sex	Age (yr.)	Migraine type	Disease duration (yr.)	Acute medicine	Prophylactic medicine
M1	M	24	MA	5	None	None
M2	F	35	MO	15	Triptan+NSAIDs	Amitriptyline
M3	M	30	MO	15	Triptan	None
M4	F	40	MO	20	Triptan+NSAIDs	None
M5	M	31	MO	10	Triptan	None
M6	F	35	MO	20	Triptan+NSAIDs	None
M7	F	36	MA	20	Triptan+NSAIDs	Propranolol
M8	F	51	MO	30	Triptan+NSAIDs	Amitriptyline
M9	F	39	MO	25	Triptan+NSAIDs	Amitriptyline
M10	F	53	MO	35	Triptan	Amitriptyline

Ten patients with migraine received triptans during the attack; the remaining received preventive drugs.

M: male; F: female; MA: migraine with aura; MO: migraine without aura

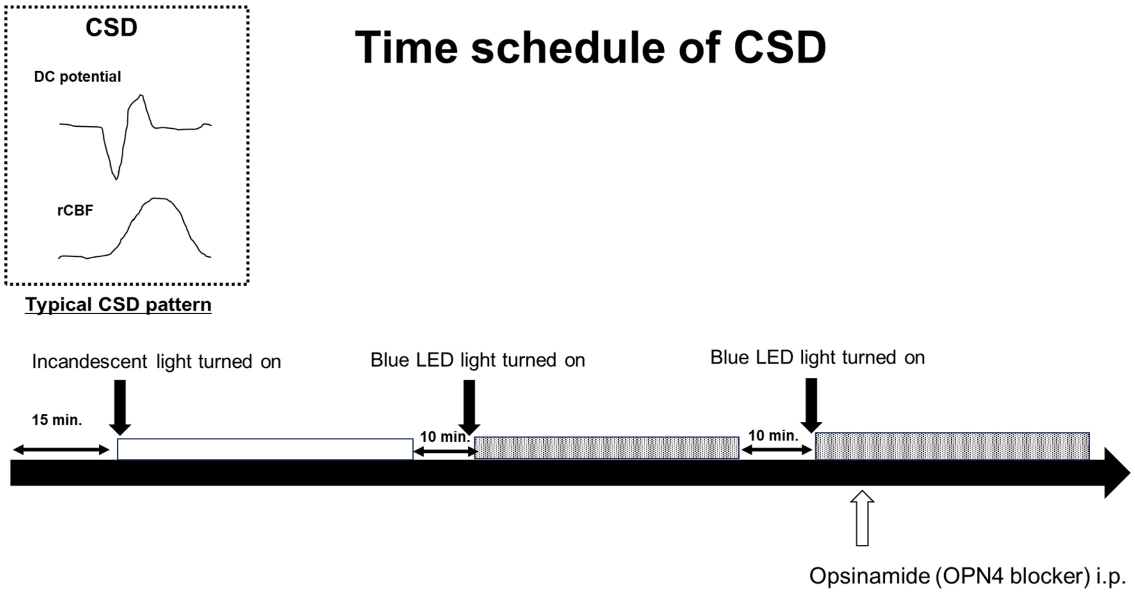

Figure S1. Experimental timeline of light/dark.

The mice were kept in the dark for 15 min prior to commencing the CSD experiments. The left eye of the mouse was irradiated with an incandescent light positioned 3 cm ahead. The CSD experiment was performed 5 min later. CSD experiments were performed by irradiating the mice with blue LED light after a 10-min dark interval. After the experiments were completed, the mice were exposed to the blue LED light again after a 10-min dark interval. Opsinamide, a melanopsin inhibitor, was administered intraperitoneally to the mice after 15 min. CSD experiment was performed for 30 min after administering opsinamide.

Video S1. Video depicting the alterations in the pupils of the patients with migraine and controls.

The upper panels depict the pupillary reactions of the controls, whereas the lower panels depict the pupillary reactions of the patients with migraine.

The opposite eyes of the controls and patients with migraine were irradiated with red and blue LED lights, and the changes in pupil diameter were observed. A small black box appeared at the bottom right of the screen when the light was illuminated.

The time taken by the miotic pupils to return to their original size after exposure to blue light in the patients with migraine was longer than that in controls.
